# An ongoing role for *Wnt* signaling in differentiating melanocytes in vivo

**DOI:** 10.1111/pcmr.12568

**Published:** 2017-03-09

**Authors:** Laura Vibert, Gerardo Aquino, Ines Gehring, Tatiana Subkankulova, Thomas F. Schilling, Andrea Rocco, Robert N. Kelsh

**Affiliations:** ^1^ Developmental Biology ProgrammeDepartment of Biology and BiochemistryCentre for Regenerative MedicineUniversity of BathBathUK; ^2^ Department of Microbial and Cellular SciencesFaculty of Health and Medical SciencesUniversity of SurreyGuildfordUK; ^3^ Developmental and Cell Biology School of Biological SciencesUniversity of California, IrvineCAUSA

**Keywords:** differentiation, melanocyte, *mitfa*, neural crest, specification, *Wnt* signaling, Zebrafish

## Abstract

A role for Wnt signaling in melanocyte specification from neural crest is conserved across vertebrates, but possible ongoing roles in melanocyte differentiation have received little attention. Using a systems biology approach to investigate the gene regulatory network underlying stable melanocyte differentiation in zebrafish highlighted a requirement for a positive‐feedback loop involving the melanocyte master regulator Mitfa. Here, we test the hypothesis that Wnt signaling contributes to that positive feedback. We show firstly that Wnt signaling remains active in differentiating melanocytes and secondly that enhanced Wnt signaling drives elevated transcription of *mitfa*. We show that chemical activation of the Wnt signaling pathway at early stages of melanocyte development enhances melanocyte specification as expected, but importantly that at later (differentiation) stages, it results in altered melanocyte morphology, although melanisation is not obviously affected. Downregulation of Wnt signaling also results in altered melanocyte morphology and organization. We conclude that Wnt signaling plays a role in regulating ongoing aspects of melanocyte differentiation in zebrafish.

**Significance**

Gene regulatory networks underlie all aspects of the development of specific cell types. While fate specification mechanisms and key genes associated with differentiation are often well studied, mechanisms leading to maintenance of the differentiated state are less well understood, yet they are crucial from a disease perspective. Positive‐feedback loops are predicted to be crucial to these maintenance mechanisms. We predicted that such a feedback loop acting on *mitfa* would be crucial for melanocyte maintenance, and provided evidence supporting this hypothesis. Here, we provide evidence for the first time that in zebrafish, ongoing Wnt signaling is likely to contribute to this feedback loop.

## Introduction

Melanocytes are a key derivative of the neural crest, and the mechanisms of melanocyte development are of major interest from developmental and stem cell biology and applied biology perspectives, with clear relevance to understanding human pigmentary disease (Aoude et al., 2015; Mort et al., 2015; Speeckaert et al., 2015; White et al., 2011; Yamaguchi and Hearing, 2014). Melanocyte specification from neural crest cells has been relatively well studied and has illuminated the underlying mechanisms of neurocristopathies like the Waardenburg syndromes (Dutton et al., 2001; Elworthy et al., 2003; Lee et al., 2000; Southard‐Smith et al., 1998). Numerous genes contributing to the differentiated melanocyte phenotype have been described, but little is known of how *stable* melanocyte differentiation is maintained, although this is likely to be significant in understanding melanoma where reversion to a more progenitor‐like state helps drive proliferation and invasiveness (Kaufman et al., 2016; White et al., 2011). Stability of differentiation is likely to be an emergent property of the state of the gene regulatory network (GRN) in differentiated melanocytes.

Studies in both mouse and zebrafish have shown that melanocyte specification depends upon the expression in neural crest cells of *microphthalmia‐related transcription factor* (*Mitf; mitfa* in zebrafish), which encodes a basic helix‐loop‐helix transcription factor (Hodgkinson et al., 1993; Lister et al., 1999; Opdecamp et al., 1997; Steingrimsson et al., 1994; Watanabe et al., 2002). *Mitf*/*mitfa* functions as the master regulator of melanocyte development as it controls all aspects of melanocyte cell biology including melanocyte survival, proliferation, morphology, and melanogenesis itself (Cheli et al., 2010; Levy et al., 2006; Lister et al., 1999; Steingrimsson et al., 2004). Transcriptional activation of *Mitf*/*mitfa* depends upon Sox10, a transcription factor of the Sry‐related HMG domain type, shown to bind directly to the *Mitf*/*mitfa* promoter (Dutton et al., 2001; Elworthy et al., 2003; Herbarth et al., 1998; Hou et al., 2006; Kelsh, 2006; Southard‐Smith et al., 1998). In addition, Wnt signaling, acting via Lef‐1‐ and *β*‐catenin‐mediated regulation of *Mitf*/*mitfa* transcription, is also required for melanocyte specification (Dorsky et al., 1998, 2000; Dunn et al., 2000; Hari et al., 2012; Jin et al., 2001; Lee et al., 2004; Takeda et al., 2000). Thus, the combined actions of early Wnt signaling and Sox10 drive expression of Mitf/Mitfa, which acts as a central node in the melanocyte GRN, activating numerous genes associated with all aspects of melanocyte differentiation (Cheli et al., 2010). Importantly, work in zebrafish has shown that a majority of neural crest cells express *mitfa* transiently, but only a subset form melanocytes (Curran et al., 2010). Thus, maintenance of *mitfa* expression in a subset of neural crest cells is an important, but under‐appreciated, aspect of melanocyte development and, indeed, likely represents a crucial part of the molecular basis for melanocyte fate commitment; cells in which *mitfa* expression is not maintained adopt alternative neural crest fates.

**Figure 1:**
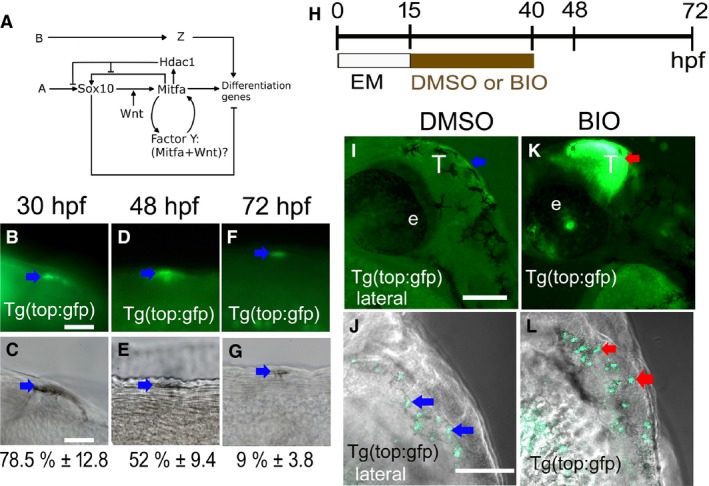
Ongoing Wnt signaling is detectable in differentiating melanocytes. (A) Diagram of working model for melanocyte core GRN (modified from Greenhill et al., 2011). Note, two inputs from Wnt signaling on *mitfa* expression, one well‐established role acting alongside Sox10 driving melanocyte fate specification, and hypothetical role explored here as part of a positive‐feedback loop also involving Mitfa itself. (B–G) Wnt signaling in differentiating melanocytes was assessed by scoring samples of melanocytes for detection of dGFP by immunofluorescence in *Tg*(*top:GFP*) transgenic embryos from 30 to 72 hpf. GFP‐positive melanocytes (arrows) are shown by immunofluorescence (B, D, F) and DIC (C, E, G) to show melanin pigment. At each stage, 30 melanocytes in each of 15 embryos were assessed for GFP expression, in the dorsal head and throughout the dorsal and lateral trunk; numbers show the percentage of melanocytes expressing GFP (mean ± SD) at corresponding stages (n = 450 per stage). (H) Schematic showing timing of BIO treatment used. (I–L) BIO treatment enhances activity of *Tg*(*top:GFP*) reporter. BIO treatment from 15 to 40 hpf dramatically enhances dGFP expression (red arrows) in the tectum of treated (K) compared with mock‐treated controls (I) and also increases dGFP signal in melanocytes of *Tg*(*top:GFP*) embryos [BIO‐treated (L) compared with mock‐treated control (J)]. All images, lateral views using confocal microscopy. EM, embryo medium; e: eye; T: tectum. Scale bars: (B–G) 10 μm; (I–L) 100 μm.

As a first step in understanding how this GRN results in stable maintenance of melanocyte differentiation, we used a systems biology approach, taking advantage of the zebrafish model to allow rapid iterative cycles of mathematical modeling and biological testing of a core melanocyte GRN (Greenhill et al., 2011). Three cycles of this approach resulted in an expanded core network that incorporated a number of hypothetical components for which we also provided experimental support (Figure 1A; Greenhill et al., 2011). One of these hypothetical components was a predicted Factor Y that enabled stabilization of melanocyte differentiation by contributing to a positive‐feedback loop regulating *mitfa* expression (Greenhill et al., 2011). We showed that while initial expression of *mitfa* is driven by Sox10*, sox10* then was gradually lost from differentiating melanocytes, being undetectable by in situ hybridisation or whole‐mount immunofluorescence by c. 50 h post‐fertilization (hpf). Importantly, we showed that continuing *sox10* expression is not required for melanocyte differentiation and indeed, may even delay this process. In the Greenhill study, we showed that Mitfa itself contributed to maintenance of *mitfa* expression, but left the identity of other possible components of the feedback loop unknown.

The canonical Wnt pathway functions via regulation of nuclear *β*‐catenin levels; Wnt protein binding to its transmembrane receptor, Frizzled, displaces glycogen synthase kinase‐3*β* (GSK3*β*) from the destruction complex, preventing degradation of *β*‐catenin in the cytoplasm, and resulting in elevated *β*‐catenin levels in the nucleus where it functions as a transcriptional activator in cooperation with a co‐activator, Lef‐1 (Barker, 2008; Kofahl and Wolf, 2010). Canonical Wnt signaling drives fate specification of melanocytes via Tcf/Lef binding to *mitfa* regulatory elements (Dorsky et al., 1998, 2000; Takeda et al., 2000; Jin et al., 2001; Hari et al., 2012; Dunn et al., 2000; Lee et al., 2004) although the relevant Frizzled receptor remains elusive (Nikaido et al., 2013). In melanocyte fate specification, Wnt signaling functions in conjunction with Sox10 to drive *mitfa* expression; in a *sox10* mutant, *mitfa* expression is not detectable and melanocytes do not form (Dutton et al., 2001), suggesting that Wnt signaling alone is insufficient to drive *mitfa* expression.

Here, we explore two possible modes by which Wnt signaling may also contribute to melanocyte maintenance: (i) by ongoing action alongside Sox10 and (ii) in a Mitfa‐dependent manner as a component of Factor Y (Figure 1A). We use a transgenic reporter line to show that Wnt signaling is active in melanocytes throughout both specification and differentiation phases. We then use small molecule stimulation of Wnt signaling showing first that it activates *mitfa* expression and is rate‐limiting for melanocyte fate specification. We then use this approach to show that stimulating Wnt signaling during melanocyte differentiation affects melanocyte morphology and arrangement according to the timing of treatment. These shape changes correlate with upregulation of *mitfa* expression levels in melanocytes. These *mitfa* expression changes are independent of Mitfa activity in the specification and early differentiation phases, but depend upon Mitfa activity during late differentiation. Conversely, using a transgenic repressor of Wnt signaling, we show that inhibition of Wnt signaling during melanocyte differentiation stages also results in melanocyte shape changes. This is consistent with our proposal that Wnt signaling contributes to a Mitfa‐dependent positive‐feedback loop that maintains melanocyte differentiation, in a manner consistent with our mathematical model of the core melanocyte GRN.

## Results

Firstly, we asked whether Wnt signaling was active in melanocytes during differentiation. We examined embryos carrying the *Tg*(*top:GFP*) reporter in which expression of a destabilised GFP (dGFP) is driven by a promoter containing four consensus LEF1‐responsive elements (Dorsky et al., 1998, 2002; Korinek et al., 1997; Van De Wetering et al., 1997). We scored a sample of melanocytes from these fish throughout melanocyte differentiation (30–72 hpf), using a combination of immunofluorescent detection of dGFP and a low dose of PTU to partially inhibit melanisation to increase sensitivity. We readily detected the activation of dGFP in the tectum as previously described (Figure 1I; Dorsky et al., 2002). We also observed, for the first time, dGFP expression in melanocytes throughout the time period examined (Figure 1B–G). At early stages, the majority of melanocytes (78.5%) had readily detectable dGFP expression, indicating that most cells at this stage were responding to a Wnt signal. At later stages, a significant proportion of cells still showed detectable dGFP expression, although by 72 hpf, the proportion of cells was substantially lower (9%), but this is likely to at least partly reflect the difficulties of detection of weak fluorescence in even partially melanised cells.

Having shown that Wnt signaling remains active in melanocytes throughout their differentiation, we then tested the hypothesis that this Wnt signaling in melanocytes might contribute to regulation of their differentiation. We utilized a well‐characterized GSK3*β* inhibitor, Bromoindirubin‐3′‐oxime (subsequently referred to as BIO), that has been used to investigate the effects of increased Wnt signaling in many model systems, including mammalian cardiomyocyte cell culture, melanoma cells (B16‐F0), normal human melanocyte (NHM) cells, mouse embryonic stem cells, and zebrafish (Alexander et al., 2014; Bellei et al., 2008, 2010; Kim et al., 2010; Moro et al., 2012; Sineva and Pospelov, 2010; Tseng et al., 2006). Consistent with its activation of Wnt signaling in our treated embryos, BIO treatment throughout a 15–40 hpf time window activated dGFP reporter expression in *Tg*(*top:GFP*) fish, both in the tectum and in the melanocytes (Figure 1K, L); importantly, this BIO treatment did not result in gross alterations to embryonic morphology.

**Figure 2:**
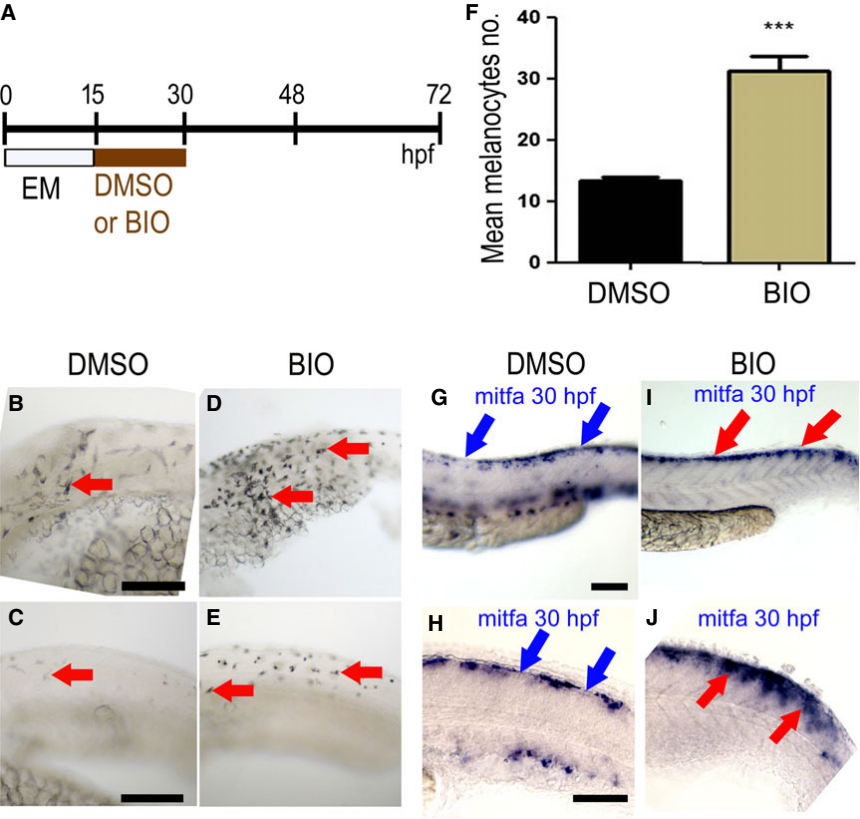
Elevated Wnt signaling in melanocyte specification time window increased numbers of melanocytes. (A) Schematic showing timing of BIO treatment or control mock treatment (DMSO alone) used. (B–E) Lateral views of the trunk of live 30 hpf zebrafish embryos showing melanocytes (red arrows); note, increased number in both anterior (D) and posterior trunk (E) of embryos treated with 5 μM BIO compared with control DMSO‐treated embryos (B, C, respectively). (F) Quantitation of increased melanocyte number in heads of BIO‐treated embryos (mean ± SD; control, 13 ± 2.1; BIO, 32 ± 5.3, P < 10^−6^) (n = 20; *** indicates significant difference between treated and mock treated; unpaired, one‐tailed t test). (G–J). Lateral views of posterior trunk (G, I) and close‐up of anterior tail (H, J) of 30 hpf zebrafish embryo in situ hybridisations to show *mitfa* expression in embryos treated with BIO (I, J) or DMSO control (G, H). In the DMSO control embryos (G, H), discontinuous *mitfa* expression is observed in the dorsal region (blue arrows show discontinuities of the signal), whereas in the BIO‐treated embryos (I, J), *mitfa* expression is continuous throughout the dorsal region (red arrows). Images show representative phenotypes (n = 20 per treatment). Scale bar: 100 μm.

Wnt signaling has been shown to drive melanocyte specification from the neural crest in both mouse and zebrafish (Dorsky et al., 1998; Hari et al., 2012). As a further positive control for the efficacy of BIO treatment in modifying Wnt signaling in our fish, we tested the prediction that BIO treatment in the correct time window would increase melanocyte specification, resulting in elevated melanocyte numbers. We used a shorter treatment phase, focused on 15–30 hpf (Figure 2A), the time when melanocyte specification is thought to occur in the head and trunk. As expected, this treatment resulted in significantly increased melanocyte number in the head of treated embryos compared with controls (Figure 2B–F). Taking advantage of the developmental gradient in these embryos, we examined the trunk and tail region of embryos fixed at the same stage to examine whether elevated melanocyte numbers might be preceded by elevated *mitfa* expression in melanocyte progenitors (premigratory neural crest cells). Consistent with our current working model of the melanocyte GRN, BIO‐treated embryos showed increased *mitfa* expression in premigratory neural crest cells compared with mock‐treated controls (Figure 2G–J). Our data suggest that the increased *mitfa* reflects increases in both the levels of *mitfa* expression within a cell and the number of *mitfa*‐expressing cells (Figure 2D, E; compared with B, C), consistent with the predicted increase in melanocyte fate specification. Taken together, our data support the conclusion that BIO treatment in a 15–30 hpf time window results in elevated melanocyte specification through increased *mitfa* expression after activation of Wnt signaling. In addition, BIO‐treated embryos show a reduction in ventrally positioned *mitfa*+ cells, suggesting a transient inhibition of melanoblast migration.

**Figure 3:**
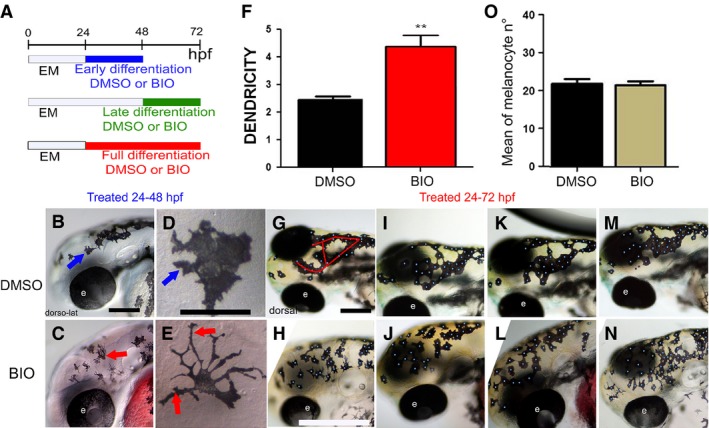
Elevated Wnt signaling in melanocyte differentiation time window alters melanocyte morphology. (A) Schematic of melanocyte differentiation BIO treatment time windows. (B–E) Activation of Wnt signaling in early differentiation period (24–48 hpf) increases melanocyte dendricity and dispersion. Lateral view at 48 hpf of head of live embryos treated with control DMSO (B) or BIO (C) from 24 to 48 hpf. Panels D and E show enlargements of head melanocytes from embryos in B and C, respectively. (F) Quantitation of dendricity: the cell roundness parameter was calculated for each melanocyte as R = P2 ⁄ 4∏A, where A is the cell area and P the cell perimeter, and 20 cells were investigated (n = 20) in 10 different embryos for each condition. A score of R = 1.0 represents a perfectly round melanocyte; increased R values represent increased dendricity. Control, R = 2.44 ± 0.43, BIO R = 4.25 ± 1.68, P = 0.004, t test result, P‐value < 0.01 (**). (G–J) Elevated Wnt signaling throughout full differentiation or during late differentiation window only affects melanocyte organization. Red line in G indicates the dorsal head melanophores which usually approximate an O‐ or U‐shaped pattern, although often with extra branches as in this specific example. Dorsolateral view at 72 hpf of heads of live embryos treated with control DMSO (G, I, K, M) or BIO (H, J, L, N) from 24 to 72 hpf shown. Note that embryos treated in only late differentiation window (48–72 hpf) show equivalent phenotype (data not shown). Embryos shown are representative of samples examined (n = 160 zebrafish embryos for each treatment). Scale bars: 100 μm (B, D, G, I).

Having validated BIO treatment as an effective method for elevating Wnt signaling, we then addressed our hypothesis that ongoing Wnt signaling might be important for melanocyte differentiation and maintenance. In this study, we divided melanocyte differentiation into early and late phases according to *sox10* expression (Figure 3A). In the early differentiation phase (24–48 hpf), *sox10* expression in melanocytes is decreasing but still detectable by WISH and immunofluorescence, whereas in the late differentiation phase (48–72 hpf), *sox10* is not detectable by WISH or immunofluorescence (Greenhill et al., 2011). We analyzed the consequences of boosting Wnt signaling via BIO treatment throughout the entire differentiation phase (24–72 hpf) and during each of the early and late phases of differentiation. Firstly, we tested whether these treatments gave any effects in terms of melanocyte cell number, focusing on the head region. In contrast to 15–30 hpf treatment, no increase in melanocyte number was observed after BIO treatment from 24 to 48 hpf (control, mean ± SD = 23.9 ± 2.01; BIO, mean ± SD = 23.1 ± 4.03, P = 0.443), suggesting that Wnt‐dependent melanocyte fate specification is complete in the head region by 24 hpf.

Next, we assessed melanocyte differentiation, looking for any changes associated with BIO treatment during the differentiation phases. In these BIO treatments, we saw distinctive morphological changes to melanocytes, but interestingly, the phenotype differed depending upon the period of BIO treatment. Embryos treated during the early differentiation time window and examined at 48 hpf displayed a dramatic increase in cell dendricity (Figure 3C, E) compared with mock‐treated controls (Figure 3B, D). To quantitate this striking phenotype, we used imagej software (Schneider et al., 2012) to estimate the roundness parameter, an established formula relating cell perimeter and cell area (Figure 3F). We found BIO treatment resulted in a significant increase in cell dendricity compared with mock‐treated controls [mean ± SD: control, 2.44 ± 0.43; BIO, 4.25 ± 1.68, P = 0.004 (unpaired, one‐tailed t test)]. Importantly, this change in cell shape at 72 hpf was not observed in 24–72 hpf (Figure 3G–N) or 48–72 hpf treatments (data not shown) suggesting that this aspect of cell differentiation was only sensitive to Wnt signaling for a period between 24 and 48 hpf, and that the shape changes were only transient. Indeed, where embryos treated in this way were examined at both 48 and 72 hpf, we saw a clear dendricity phenotype at 48 hpf, but this had recovered by 72 hpf (data not shown). In contrast, embryos treated with BIO from 24 to 72 hpf showed disorganized melanocytes in the dorsal head; cells were more dispersed, disrupting the ‘U’‐ or ‘O’‐shaped organization typical of control embryos (Figure 3G–J). Treatment during the late differentiation phase alone (i.e., from 48 to 72 hpf) resulted in a similar phenotype (data not shown). A multidistance spatial cluster analysis using Ripley's K Function (Ripley, 1977) was performed using spatstat package in r (Baddeley and Turner, 2005) to quantify the decrease in patterned organization of melanocytes in the dorsal head using comparable pictures of DMSO‐ and BIO‐treated fish (Figure S1). We found that the Ripley's K function confirms the non‐random organization of melanocytes in dorsal heads of DMSO‐treated embryos (scale of analysis of r = 35 pixels). Indeed, at this scale of analysis, for eight of the ten embryos, the observed K value was smaller than the expected K value if cells were randomly spread (Figure S1A–C, E, G, H, J). The result of the Ripley's K analysis performed on BIO‐treated embryos showed that, at r = 35, the observed K values were found within the lower confidence envelop (in the gray area) of the graph meaning that cells were randomly spread in 90% of the cases in nine of ten embryos (Figure S1K–T). Conversely, 80% of the DMSO population shows a non‐random cell organization and 10% of the BIO‐treated population shows a non‐random cell organization. A z‐test was then performed to compare the two populations for cellular spatial organization using the results of this analysis (at r = 35, comparing ‘random’ or ‘not‐random’); the two populations were significantly different for this parameter (P‐value = 0.00164, P < 0.01). We conclude that treating embryos with BIO in the early phase of differentiation led to changes in cell shape, whereas ongoing BIO treatment resulted in changed melanocyte arrangement.

**Figure 4:**
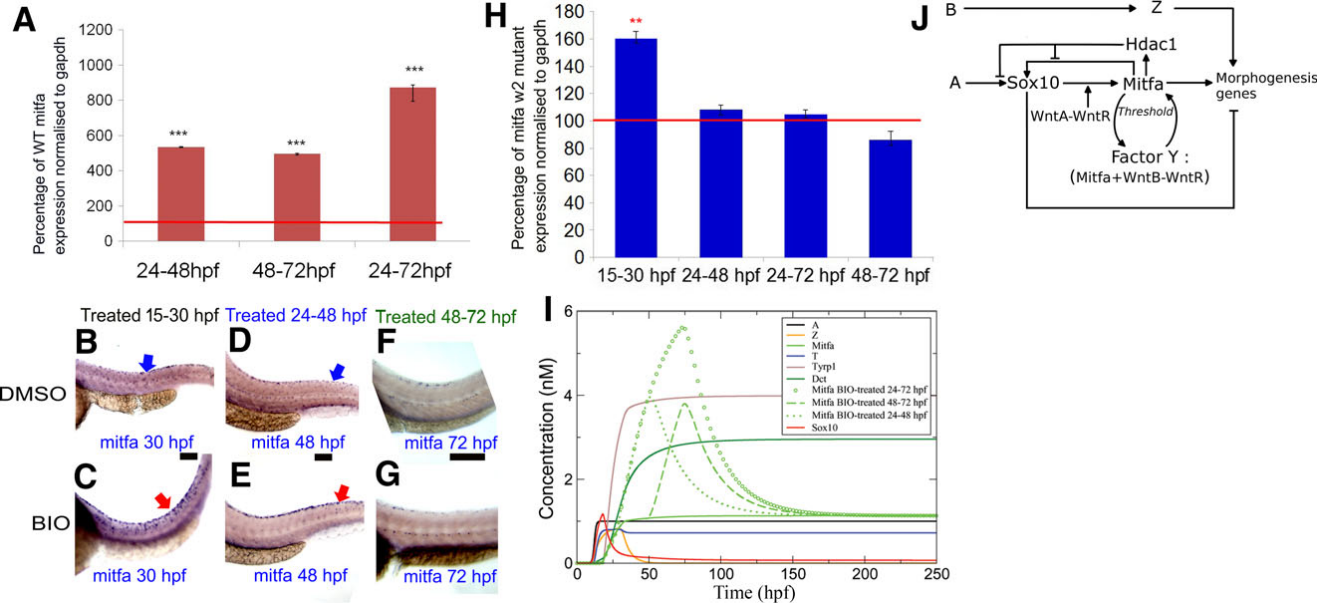
Positive feedback between Wnt signaling and Mitfa during late differentiation phase of melanocyte development. (A) Quantitation of *mitfa* expression by RT‐qPCR showing significantly increased levels in BIO‐treated embryos compared with untreated controls; data are expressed as percentage of mock‐treated controls [control *mitfa* level = 100% transcripts (red line on graph)]. Gene expression was investigated in 10 samples consisting of the trunk and tail of 50 embryos for each condition and in triplicate, after normalization to the stable expression of a reference house‐keeping gene, *gapdh*. All treatments caused significant increase of *mitfa* expression [t test, two‐tailed: 24–48 hpf, 535 ± 7.1 (P = 0.00087); 48–72 hpf, 496 ± 9.8 (P = 0.0085); 24–72 hpf, 872 ± 32.1 (P = 0.000092). ***P‐value < 0.001]. (B–G) Activating Wnt signaling after 48 hpf does not affect *mitfa* expression in *mitfa* ^*w2*^ mutant embryos during melanocyte differentiation. Lateral views of trunk showing in situ hybridisation for *mitfa* expression in *mitfa* mutant fish. *mitfa* mutant embryos treated with BIO in the melanocyte specification phase (15–30 hpf) (C) showed increased *mitfa* expression (purple) compared to DMSO‐treated embryos (B). Similarly, *mitfa* mutant embryos treated with BIO in the melanocyte early differentiation phase (24–48 hpf) (E) showed increased *mitfa* expression in tail dorsal stripe melanocytes compared to DMSO‐treated embryos (D). In contrast, *mitfa* mutant embryos treated with BIO throughout the late melanocyte differentiation phase (48–72 hpf) (G) did not show increased *mitfa* expression compared to DMSO‐treated embryos (F) at 72 hpf; 40 zebrafish embryos were investigated for each condition; BIO‐treated and matched control embryos were processed for in situ hybridisation in parallel and under identical conditions. (H) Quantitation of *mitfa* expression by RT‐qPCR showing significantly increased levels in BIO‐treated embryos at 30 hpf compared with untreated controls but not at 48 hpf nor at 72 hpf; data are expressed as percentage of mock‐treated controls [control *mitfa* level = 100% transcripts (red line on graph)]. Gene expression was investigated in triplicate, after normalization to the stable expression of a reference housekeeping gene, *gapdh*. Only 15–30 hpf treatment caused significant increase of *mitfa* expression [t test, two‐tailed: 15–30 hpf, 160.39 ± 7.27 (P = 0.0020); 24–48 hpf, 108.15 ± 7.9 (P = 0.0675); 24–72 hpf, 104.6 ± 8.36 (P = 0.544); 24–72 hpf, 86.14 ± 8.96 (P = 0.0831). **P‐value < 0.01]. (I) The effect of the three BIO treatments applied in the 24–48, 48–72, and 24–72 hpf time windows is shown on Mitfa only as compared to the case of no treatment. The 24–48 and 48–72 hpf BIO treatments induce an approximately equal increase of Mitfa as measured at the end of the treatment. (J) Revised GRN model. Scale bars: 100 μm.

Our working model of the melanocyte GRN predicted that *mitfa* transcription would be increased in response to activation of Wnt signaling. Using quantitative RT‐qPCR, we saw a BIO‐dependent increase in *mitfa* transcript levels after each of the treatment windows (Figure 4A). *mitfa* expression was increased by around fivefold in embryos treated from both 24 to 48 hpf and 48 to 72 hpf whereas a ninefold increase was observed in embryos treated from 24 to 72 hpf. Combining these data with the melanocyte phenotype changes observed above, we suggest that elevated *mitfa* expression can have distinct effects depending upon whether cells are in a plastic progenitor state (fate specification phase) or are committed to the melanocyte fate (differentiation phase).

Our model (Figure 1A) proposes two modes for Wnt action, one in conjunction with Sox10 and thus unlikely to be significant beyond 48 hpf when *sox10* expression in melanocytes is lost (Greenhill et al., 2011), and one as Factor Y, where it would function as part of a positive‐feedback loop with Mitfa, and thus would be dependent upon active Mitfa. To test whether the increase in *mitfa* expression observed in BIO‐treated embryos was (i) specific for melanocytes and (ii) Mitfa dependent, we used whole‐mount in situ hybridisation and RT‐qPCR to assess *mitfa* expression in BIO‐treated *mitfa* ^*w2*^ mutants (Figure 4B–H). The *mitfa* ^*w2*^ mutant is a functional null allele, but expression of *mitfa* transcripts can be assessed by in situ hybridisation (Johnson et al., 2011). In all cases, *mitfa* expression was restricted to cells with the morphology and distribution consistent with them being neural crest or melanocyte lineage cells, consistent with a cell‐autonomous response. Importantly, BIO treatment of *mitfa* ^*w2*^ mutants in a 15–30 hpf window (i.e., during cell specification, when *sox10* is relatively strongly expressed in the melanocyte lineage) resulted in increased *mitfa* expression (Figure 4H), most clearly seen as increased numbers of cells expressing relatively high levels of *mitfa*, at 30 hpf compared with mock‐treated embryos (Figure 4B, C). A 24–48 hpf BIO treatment, assessed at 48 hpf, showed an elevated level of *mitfa* expression in *mitfa* ^*w2*^ mutants, at least in the dorsal tail (Figure 4D, E). In contrast, BIO treatment in a 24–72 (data not shown) or 48–72 hpf window (Figure 4F, G), assessed at 72 hpf, did not show elevated *mitfa* transcription in these *mitfa* ^*w2*^ mutants (Figure 4H), suggesting that the Wnt‐mediated effect is Mitfa dependent. Quantitation of *mitfa* expression levels using RT‐qPCR confirmed the significant increase in *mitfa* expression after 15–30 hpf treatment even in *mitfa* ^*w2*^ mutants, and the absence of a significant response after the 24–72 and 48–72 hpf time windows. Our quantitative assay did not detect the change after a 24–48 hpf time window: although consistently detectable by the semi‐quantitative whole‐mount in situ hybridisation technique, which has the advantage of assessing individual cells in different spatial domains, this signal is apparently masked by the general signal coming from the head and trunk. We conclude that Wnt inputs on *mitfa* expression act initially through the Sox10‐dependent mechanism, but then switch to functioning through a Sox10‐independent, Mitfa‐dependent positive‐feedback loop.

**Figure 5:**
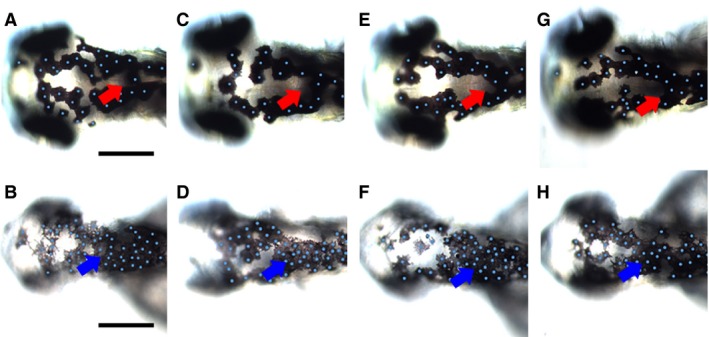
Inactivation of Wnt signaling during melanocyte differentiation enhances melanocyte clustering in posterior dorsal head. *Tg*(*hsp70l:tcf3‐deltaC‐GFP*) embryos were heat‐shocked at 29, 36, 48, and 62 hpf and observed at 73 hpf (B, D, F, H) and compared to control non‐heat‐shocked transgenic *Tg*(*hsp70l:tcf3‐deltaC‐GFP*) (A, C, E, G). Note the enhanced clustering of melanocytes in the posterior part of the dorsal head in treated embryos (blue arrowheads); the normal distribution in this area in untreated controls is indicated by red arrows. Heat shock activation of Wnt inhibition consistently resulted in smaller heads and eyes. Scale bars: 100 μm.

As a complement to these gain‐of‐function studies, we tested the effects of loss of Wnt signaling activity during melanocyte differentiation using a conditional activation of the dominant‐negative T‐cell Factor 3 (Tcf3) in transgenic zebrafish carrying *Tg*(*hsp70l:tcf3‐deltaC‐GFP*) (Martin and Kimelman, 2012). As before, we tested the functionality of our experimental system by inducing heat shock during the specification phase of melanocyte development, from 15 to 28 hpf, and assessing first activation of GFP fluorescence and expression of Wnt signaling targets such as *mitfa*, by RT‐qPCR. Both activation of GFP and downregulation of *mitfa* expression were observed after heat shock (data not shown). To assess the requirement for ongoing Wnt signaling in melanocyte differentiation, we heat‐shocked embryos at each of four timepoints, 29, 36, 48, and 62 hpf, before assessing melanocyte phenotypes in the dorsal head at 73 hpf. Two populations of embryos could be distinguished by the level of activation of GFP, one where embryos showed strong GFP fluorescence and the second population showing weak GFP fluorescence; we note that in almost all cases, melanocytes were abundant, indicating that survival of melanocytes was usually not affected, although in a subset of the fish exhibiting strong GFP, melanocyte number was visibly reduced. In the first population (strong inhibition of Wnt signaling), melanocyte melanisation, apparent morphology, and patterning were all abnormal, but as the gross anatomy of the embryos was also affected, these melanocyte phenotypes were not quantitated. However, we noticed that a subset of melanocytes in each fish was abnormally rounded, melanisation was always reduced, and these cells were abnormally patterned (Figure S2). In the second population (weak inhibition of Wnt signaling), melanocyte morphology was more normal, but their patterning was modified compared to non‐heat‐shocked siblings (Figure 5). We observed enhanced clustering of melanocytes, particularly in the posterior region of the dorsal head (Figure 5B, D, F, H, blue arrowheads). Again, we used multidistance spatial cluster analysis using Ripley's K Function (Ripley, 1977) to quantify the change in patterned organization of melanocytes in the dorsal head using comparable pictures (Figure S3). This analysis showed that whereas 90% of the non‐heat‐shocked *Tg*(*hsp70l:tcf3‐deltaC‐GFP*) embryos showed ‘non‐random’ pattern, 70% of the heat‐shocked *Tg*(*hsp70l:tcf3‐deltaC‐GFP*) embryos showed loss of organization (‘random’ cell organization—the combination of the clustered melanocytes in the posterior head and the unclustered melanocytes in the anterior head are together scored by the algorithm as more randomly organized using this scale of analysis)(scale of analysis of r = 50 pixels). A z‐test comparing the two populations for cellular spatial organization using the results of this analysis (at r = 50, comparing ‘random’ or ‘not‐random’) showed they were significantly different for this parameter (P‐value = 0.00244, P < 0.01). We conclude that inactivating Wnt signaling during melanocyte differentiation in *Tg*(*hsp70l:tcf3‐deltaC‐GFP*) using heat shock led to significantly increased clustering of melanocytes in the posterior part of the dorsal head. We note that this loss‐of‐function phenotype is complementary to the gain‐of‐function phenotype using drug treatment, suggesting that Wnt signaling has a consistent impact on melanocyte differentiation in zebrafish in vivo.

We used the mathematical modeling approach we developed before (Greenhill et al., 2011) to assess the effects of including Wnt signaling (Figure S4A). We incorporated Wnt signaling in both fate specification and differentiation phases by assuming Wnt expression to activate *β*‐catenin/Lef‐1 (abbreviated as T), coupled in turn with an AND gate to Sox10 during specification, and by another AND gate to Mitfa during commitment. For such a highly migratory cell type, melanocyte fate specification (in/near the dorsal neural tube) and melanocyte differentiation (during dispersion and in post‐migratory locations) are usually anatomically distinct and, given the complexity of Wnt expression patterns, thus are intuitively likely to involve distinct Wnt family members. Thus, we postulated two independent Wnt signals, one (WntA) acting in an early, but transient, time window lasting to approximately 30 hpf, and another (WntB) beginning at that latter timepoint and extending throughout differentiation. We also assumed a threshold level of T required to trigger a response from the AND gate between Sox10 and T (Figure S4A, B), and a threshold level of Mitfa to avoid accidental activation of the Mitfa feedback loop (see Greenhill et al., 2011). As in Greenhill et al. (2011), one of the key outcomes used to judge the appropriateness of the subsequent simulations was that *mitfa* expression initially rose, and then was maintained at a substantial level. Indeed, under normal conditions, our revised model results in expression profiles comparable to those of the original Greenhill Model C (Greenhill et al., 2011). Moreover, modeling the effects of mutating the two Wnt signals independently behave as expected intuitively (Figure S4C, D). In particular, in the absence of the first Wnt signal, WntA, melanocyte specification fails and *mitfa* expression remains absent (Figure S4C); likewise, in the absence of the second Wnt signal, WntB, *mitfa* expression peaks transiently before disappearing rapidly, so that maintenance of the differentiated state fails (Figure S4D). Currently, Wnt8 has been proposed as an inducer of neural crest in zebrafish (Lewis et al., 2004), but knowledge of the Wnt signals underlying melanocyte development in fish is very limited, although Wnt8 has been shown not to be critical (Dorsky et al., 1998; Lewis et al., 2004); a testable prediction of our modeling is that two distinct Wnt signals drive early melanocyte fate specification versus later maintenance of differentiation.

We then assessed in silico the effect of increasing Wnt signaling strength in each of the specification and (early, late, and full) differentiation phases. We did this by modifying the amount of WntA and WntB driving expression of T, and simulating accordingly the GRN response. We modeled the derepression of the Wnt signal due to the action of the BIO treatment as a linear increase in time of WntA/B. As a consequence, we expected the elevated expression of *mitfa* induced by BIO to be proportional to the duration of the BIO treatment. Indeed, by choosing parameter values compatible with values in the literature (see Methods), the simulations show a relative increase of Mitfa expression in response to BIO compatible with the experimental data (Figure 2). In particular, we show that when BIO is applied for the same amount of time (namely in the time windows 24–48 and 48–72 hpf), the increase in *mitfa* is approximately the same. In contrast, when BIO is applied between 24 and 72 hpf, our simulations show a larger *mitfa* increase, roughly comparable with the observed linear dependency. Thus, our revised model with its more precise delineation of the roles of Wnt signals at different phases in melanocyte development continues to correspond well to the key experimental observations in vivo.

Our observation that elevated Wnt signaling in the specification phase results in more cells becoming melanocytes is readily accounted for by the model through the threshold on T. The likelihood of a *mitfa‐*expressing cell maintaining *mitfa* expression and thus differentiating into a melanocyte depends on T being above this threshold in the specification phase. In a population of cells, we expect stochastic variations in the levels of WntA (and hence T) will result in this condition not being satisfied in some cells. If BIO is applied early, T levels are enhanced, and therefore during the specification phase (i.e., prior to commitment), the proportion of cells proceeding successfully toward the melanocyte state is predicted to increase. In contrast, if BIO is only applied later, T levels during the earlier specification window are unaffected, so no change in the final number of melanocytes is predicted, in agreement with our experimental data.

## Discussion

We conclude that in addition to the established role for Wnt signaling in melanocyte specification from the neural crest, Wnt signaling has an ongoing influence on melanocyte differentiation at least until 72 hpf. Using a transgenic reporter strain, we demonstrated that Wnt signaling remained detectable in differentiated melanocytes until 72 hpf at least. We then used BIO to assess the effects of increased Wnt signaling on melanocyte development in zebrafish embryos, showing that such treatment resulted in elevated *mitfa* expression in the neural crest and melanocyte lineage cells. Interestingly, we observed defects in melanocyte morphology with an increase in cell dendricity at 48 hpf and altered cell organization at 72 hpf. We complemented these gain‐of‐function studies with a loss‐of‐function approach, using a conditional dominant‐negative transgenic line to inhibit Wnt signaling activity during melanocyte differentiation phases. Importantly, our data showed that this resulted changes in melanocyte morphology (when dnTcf3 strongly expressed), resulting for example in enhanced clustering of melanocytes in the posterior head. This study demonstrates for the first time a function for Wnt signaling in zebrafish melanocytes, beyond the well‐known role in melanocyte fate specification, showing that it is important for multiple aspects of melanocyte differentiation. We note that our data indicate that the mode of Wnt signaling input changes from a Mitfa‐independent/Sox10‐dependent early role to a Mitfa‐dependent/Sox10‐independent later role, suggesting that Wnt signaling at late stages becomes incorporated into the melanocyte maintenance (Factor Y) role postulated in our earlier work. We have revised our working model of the core GRN of melanocyte development in zebrafish to incorporate these new data (Figure 4J). It is likely that Wnt signaling has a similar role in mammalian melanocytes as GSK3*β* inhibition results in upregulation of *MITF* expression and differentiation in normal human melanocytes, at least in cell culture (Bellei et al., 2008).

Although we show here that Wnt signaling continues to influence melanocyte differentiation, our gain‐of‐function experiments do not cause obvious effects on melanisation. However, we note that our modeling data (Figure S4E, F) indicate that this may be quantitatively relatively subtle (e.g., for *dct* in curves shown), so our failure to detect such an effect in vivo may be simply due to lack of sensitivity in our assay. A study of normal human melanocytes in tissue culture showed that GSK3*β* inhibition stimulated melanogenesis through activation of *MITF* (Bellei et al., 2008). In contrast, in our loss‐of‐function experiments, those embryos showing highest levels of GFP and in which Wnt signaling was presumably most inhibited showed a pronounced reduction in both melanin levels and defects in apparent cell morphology. Thus, it seems that Wnt signaling is important for all aspects of melanocyte differentiation, consistent with its effects on expression of *mitfa*.

The role for Wnt/*β*‐catenin signaling in melanocyte specification through induction of *Mitf* expression is conserved in fish and mammals (Dorsky et al., 2000; Takeda et al., 2000). Our work showing that inhibition of GSK3‐*β* using BIO stimulates melanoblast specification (*mitfa* expression) adds further support to this conclusion. Importantly, no increase in melanocyte cell number in the dorsal head was observed after 24–72 hpf treatments suggesting that melanocyte specification from neural crest cells does not extend much beyond 24 hpf. Furthermore, our time window studies showed that treatment from 15 to 30 hpf was sufficient to generate elevated numbers of head melanocytes that persist until at least 72 hpf, whereas treatment in a 24–48 hpf window did not. These data indicate, for the first time to our knowledge, that commitment to the melanocyte fate in zebrafish (at least for melanocytes in the head region that arise in the embryo) has occurred by c. 30 hpf. As explained above, melanocyte specification reflects *maintenance* of *mitfa* expression in NCCs, so our data suggest that this process is limited by Wnt signaling.

Our data demonstrate intriguing Wnt‐dependent effects on melanocyte morphology and organization. An in vitro study of normal human epidermal melanocytes demonstrated that increased and decreased levels of active *β*‐catenin resulted in increased and decreased cell dendricity, respectively (Kim et al., 2010). They further showed that this resulted from activation of distinct downstream modulators, PKCf and PKCd, respectively (Kim et al., 2010). They showed that overexpression of PKCf resulted in the decrease of protein levels for Rac1 and Cdc42 and consequently a reduction of dendrite formation in melanocytes, while overexpression of PKCd led to increase of Rac1 and Cdc42 and an increase in melanocyte dendrites. We show here that elevated Wnt signaling results in increased *mitfa* expression and increased dendricity, suggesting a causal link between *mitfa* expression levels and cell shape. This idea is strongly supported by earlier studies in Xenopus embryos showing that increased *X‐mitfa* expression resulted in increased melanocyte dendricity and cell dispersal (Kawasaki et al., 2008; Kumasaka et al., 2005). Further studies will be required to test whether Wnt signaling in zebrafish causes increased melanocyte dendricity through a pathway of *mitfa*‐dependent activation of PKCd, elevated levels of activated Rac and Cdc42, and consequent modifications of the actin cytoskeleton.

In summary, our work defines two phases of Wnt signaling involvement in zebrafish melanocyte development in vivo, both of which are likely conserved in mammals. Recent analysis showed that the two transcription factors Tfap2a and Tfap2e are required for early melanocyte differentiation, through both maintenance of *kit* expression and another mechanism still to be defined (Van Otterloo et al., 2010). It will be of interest to assess whether these roles for Tfap2 factors extend to ongoing maintenance of the differentiated state and, if so, the extent to which Wnt signaling and Tfap2/Kit function are integrated in this system.

## Methods

### Fish husbandry

Wild‐type (AB) and transgenic *Tg*(*‐7.2sox10:EGFP*) (Carney et al., 2006) and *Tg*(*top:GFP*) (Dorsky et al., 2002) and mutant *mitfa* ^*w2*^ (Lister et al., 1999) zebrafish, *Danio rerio*, were kept in the aquarium at the University of Bath. *Tg*(*hsp70l:tcf3‐deltaC‐GFP*) (=w26Tg) fish (Martin and Kimelman, 2012) were kept in the facilities of the Department of Developmental and Cell Biology, School of Biological Sciences at the University of California, Irvine. Natural crosses were set up overnight and embryos collected in the morning. Embryos were placed in embryo medium and grown at 28.5°C. They were staged according to Kimmel et al. (1995). Where embryos were to be manipulated between laying and hatching, we used Watchmakers' No5 forceps to dechorionate the embryos. Embryos older than 15 hpf which were to be manipulated in any way were anaesthetised with Tricaine (ethyl 3‐aminobenzoate methanesulphonate, 4 g/l stock, final concentration approximately 0.2% v/v). Where appropriate, melanisation was inhibited using PTU (1‐phenyl‐2‐thiourea) from 24 hpf at a final concentration of 0.0015% in embryo medium. All experiments complied with institutional and national animal welfare laws, guidelines, and policies. Procedures involving fish older than 5 dpf were undertaken under license from the UK Home Office.

### BIO (2′*Z*,3′*E*)‐6‐bromoindirubin‐3′‐oxime) treatment

BIO (2′*Z*,3′*E*)‐6‐bromoindirubin‐3′‐oxime): GSK3*β* inhibitor (Calbiochem; Merck Chemicals Ltd., Nottingham, UK) was stored as a 10 mM stock solution in DMSO. Ten wild‐type or *mitfa* ^*w2*^ mutant embryos were placed in triplicate into 6‐cm petri dishes containing 8 ml embryo medium. All embryos were dechorionated before 10 mM BIO (361550 GSK‐3 Inhibitor IX, Calbiochem) in DMSO was added to each experimental dish to a final concentration of 5 μM; an equivalent volume of DMSO was added to the control dishes. Embryos were incubated under standard conditions from 15 to 30, 24 to 48, 48 to 72, or 24 to 72 hpf.

### Heat‐shock experiment

*Tg*(*hsp70l:tcf3‐deltaC‐GFP*) transgenic embryos (Martin and Kimelman, 2012) were heat‐shocked at 39°C for 12 min at four timepoints (29, 36, 48, and 60 hpf) during melanocyte differentiation; after heat shock, embryos were assessed for GFP expression and left to develop at 28.8°C in incubator. Embryos were assessed for melanocyte differentiation defects at 73 hpf.

### Whole‐mount in situ hybridisation

RNA in situ hybridisation was performed according to Thisse and Thisse (2008) except probes were not hydrolyzed and embryos were incubated at 68°C in hybridization steps. Probes used were *sox10* (Dutton et al., 2001), *mitfa* (Lister et al., 1999), and *xdh* (Parichy et al., 2000). (Plasmid and probe generated by T. Chipperfield and C. Nelson).

### RNA extraction and cDNA synthesis

RNA was extracted from samples consisting of 50 whole embryos of each condition using TRI REAGENT (Sigma‐Aldrich, Irvine, UK, T9424) according to manufacturer's instructions and purified and precipitated by phenol/chloroform/isoamylalcohol (25:24:1). The RNA pellet was washed in 1 ml of 75% ETOH by inverting the tube gently. A 1 μl sample was assessed for purity, integrity by gel electrophoresis, and concentration was measured spectrophotometrically. When required, samples were stored at −80°C for not more than a month. For experiment using WT embryos, first‐strand cDNA was synthesised using the Invitrogen First‐strand cDNA synthesis kit with Superscript III and oligodT (Promega, Southampton, UK), 0.5 μl random hexamers (250 ng/μl) (Promega), 5 μl dNTPs (2 mM) (Promega), and 7.5 μl RNA (1 μg of total RNA) were first mixed and incubated for 5 min at 65°C and then at least 1 min at 4°C. Secondly, 4 μl of 5× first‐strand buffer (Invitrogen, Paisley, UK), 1 μl of 5 mM DTT, 1 μl RNase out (Invitrogen), and 1 μl superscript III (Invitrogen) RTase/Mμl were added to the previous mix and left 5 min at 25°C, 60 min at 50°C, 15 min at 70°C. Finally, samples were diluted 1:5 and concentrations were measured spectrophotometrically. For experiments using mitfa^w2^ mutant embryos, RNA extraction was performed by Direct‐zol™ RNA MiniPrep (Zymo Research, Cambridge, UK) and the iScript™ Advanced cDNA Synthesis Kit (Bio‐Rad Laboratories Ltd., Watford, Hertfordshire, UK) was used for reverse transcription of 1 μg RNA in 20 μl reaction according to manufacturer's protocol.

### Real‐time quantitative PCR

Real‐time quantitative PCR was performed in triplicate using SYBR Green I PCR Master Mix (Roche, West Sussex, UK) for WT embryos, and a Lightcycler II machine (Roche) was used according to the manufacturer's instructions. For *mitfa* ^*w2*^ mutant embryos, the Fast SYBR^®^ Green Master Mix (Thermo Fisher, Paisley, UK) was used and samples were run in triplicate using the StepOne™ System (Thermo Fisher) according to manufacturer's instructions. Standard curves for both *gapdh* and *mitfa* primers demonstrated nearly 100% efficiency (98.7% and 98.5%, correspondently). Primers were designed spanning an intron using primer3 plus software (http://www.bioinformatics.nl/cgibin/primer3plus/primer3plus.cgi.). The following primers were used:

*gapdh*: forward 5′ACCAACTGCCTGGCTCCT3′, reverse 5′TACTTTGCCTACAGCCTTGG3′;
*mitfa*: forward 5′CTGGACCATGTGGCAAGTTT3′, reverse 5′GAGGTTGTGGTTGTCCTTCT3′.

Gene expression was normalized against zebrafish *gapdh* expression in wild‐type embryos. RT‐qPCR data were analyzed using the (ΔΔCt) method (Livak et al., 1998). Student's t test was performed using GraphPad Prism 5.0. In all tests, difference was considered significant if P < 0.001.

### Immunofluorescence

Embryos for immunofluorescent detection were processed following the protocol proposed by (Ungos et al., 2003). Embryos were fixed in 4% paraformaldehyde in PBS (phosphate‐buffered saline; Oxoid, Basingstoke, Hampshire, UK) overnight at 4°C. They were washed three times for 5 min in PBTriton (0.1% Triton X‐100 Sigma‐Aldrich in PBS) and three times for 1 h in MilliQ water. Embryos were incubated in block solution (1% DMSO, 5% Goat/Sheep serum diluted in PBTriton) for 2–3 h. They were then incubated at room temperature overnight in polyclonal mouse serum primary antibody (polyclonal IgG rabbit anti‐GFP primary antibody; Invitrogen, A11122) diluted 1:500 in block solution. Embryos were washed in PBTriton once briefly and three times for 1 h. They were incubated overnight at 4°C in Alexa Fluor 488 fluorescent anti‐rabbit secondary antibody (polyclonal IgG Alexa Fluor 488 donkey anti‐rabbit; Invitrogen, A21206) diluted 1:200 in block solution. Embryos were then washed once briefly and three times for 30 min in PBTriton. They were stored in 50% glycerol for imaging and storage.

### Microscopy

Fish were mounted between bridged coverslips in methylcellulose for live embryos, anesthetized with 0.003% MS222 (Sigma), and in 80% glycerol for fixed embryos. Embryos were photographed using a Spot digital camera (Leica, Manchester, UK) mounted on an Eclipse E800 microscope (Nikon UK Limited, Kingston upon Thames, Surrey, UK) or Axioplan 2 microscope (Zeiss, Cambridge, UK) with DIC optics using a Spot digital camera mounted on a MZ12 microscope (Leica) with epi‐illumination or Nikon sight DS‐U1 camera (Nikon UK Limited). To minimize the effects of the developmental gradient along the body axis, melanocyte counts were performed on the head only, from the anteriormost region (forebrain) to the rear of the otic vesicle (posterior hindbrain); melanocytes were counted only in the region dorsal to the CNS (i.e., the developing dorsal stripe).

### Dendricity measurement

Dendricity was calculated as *R* (*R *= *P*2/4 pA, where A is the cell area and P the cell perimeter), and cell perimeter and area were measured using imagej software from images of cells taken using Eclipse E800 microscope (Nikon UK Limited).

### Ripley's K function analysis

To quantify and compare melanocyte organization in DMSO‐treated embryos and BIO‐treated embryos, the multidistance spatial cluster analysis, based on Ripley's K Function, was performed using r statistical software (using packages spatstat and dplyr (R Core Team (2012)). Melanocyte positions were determined on the dorsal head of ten treated 72 hpf embryos for each conditions (DMSO and BIO treated) using comparable pictures. *x* and *y* coordinates for each melanocyte's position were determined using ImageJ. This data were then used to perform the Ripley's K function analysis which allows testing for random partition of the cells.

A z‐test allowed comparison of the two populations for cellular spatial organization using the results of this analysis; the result was considered significant if P < 0.01.

### Statistics

Data were analyzed by prism 3.0 software graphpadprism 5.0 using unpaired t test, *α* = 0.05%, n = 20, and fd = (*x*−2), with *x* = number of embryo tested.

### Mathematical modeling

Following the scheme of Figure S4A and indicating the molecular species with their initials (except Tyrp1, indicated with *T*_*y*_), we describe the behavior of the GRN by the following set of ordinary differential equations:

$$\frac{dZ}{dt}=g_{Z}\frac{B}{K_{B}+B}-d_{Z}Z$$

$$\frac{dS}{dt}=g_{S}\left[\frac{\Phi_{2}A\left(\Omega_{1}\Omega_{2}+\Omega_{1}H\right)+\Omega_{2}M\left(\Phi_{1}\Phi_{2}+\Phi_{1}H\right)+\Omega_{2}M\Phi_{2}A}{\left(\Phi_{1}\Phi_{2}+\Phi_{2}A+\Phi_{1}H\right)\left(\Omega_{1}\Omega_{2}+\Omega_{2}M+\Omega_{1}H\right)}\right]-d_{S}S$$

$$\frac{dM}{dt}=g_{M}\left[\frac{\left(\Gamma_{2}S+\Gamma_{1}S^{2}\right)\tilde{T}}{\left(\Gamma_{12}+\Gamma_{2}S+\Gamma_{1}S^{2}\right)\left(K_{T}+\tilde{T}\right)}\left(1-\frac{\tilde{M}T}{\left(K_{T}+T\right)\left(K_{M}+\tilde{M}\right)}\right)+\frac{\tilde{M}\tilde{T}}{\left(K_{T}+\tilde{T}\right)\left(K_{M}+\tilde{M}\right)}\right]-d_{M}M$$

$$\frac{dT}{dt}=g_{T}\frac{K_{WB}W_{A}\left(t\right)+K_{WA}W_{B}\left(t\right)+W_{A}\left(t\right)W_{B}\left(t\right)}{\left(K_{WB}+W_{B}\left(t\right)\right)\left(K_{WA}+W_{A}\left(t\right)\right)}-d_{T}T$$

$$\frac{dH}{dt}=g_{H}\frac{M}{K_{M1}+M}-d_{H}H$$

$$\frac{dT_{y}}{dt}=g_{Ty}\frac{M}{K_{M2}+M}-d_{Ty}T_{y}$$

$$\frac{dD}{dt}=g_{D}\frac{K_{Z}M+K_{M3}Z+MZ}{\left(K_{Z}+Z\right)\left(K_{M3}+M\right)}\frac{K_{S}}{K_{S}+S}-d_{D}D$$

$$\text{with}:\left\{\begin{array}{c} \tilde{M}=M\Theta \left(M-\overset{¯}{M}\right)\\ \tilde{T}=T\Theta \left(T-\overset{¯}{T}\right) \end{array}\right.,\;\left\{\begin{array}{c} W_{A}\left(t\right)=W_{A}\left(\Theta (t-30\;h)-\Theta (t-15\;h)\right)G_{\mathrm{BIO}}\\ W_{B}\left(t\right)=W_{B}\Theta \left(t-30\;h\right)G_{\mathrm{BIO}} \end{array}\right.$$

$$\text{and}:\Theta \left(x\right)=\left\{\begin{array}{cc} 0 & x<0\\ 1 & x\geq 0 \end{array}\right.,\;G_{\mathrm{BIO}}=\left\{\begin{array}{cc} 1 & \text{untreated}\\ \left(t-t_{\mathrm{BIO}}^{S}\right)\left(\Theta \left(t-t_{\mathrm{BIO}}^{E}\right)-\Theta \left(t-t_{\mathrm{BIO}}^{S}\right)\right) & \text{BIO‐treated} \end{array}\right.$$

All equations have been derived from the network scheme in Figure S4A by applying the rules explained in Greenhill et al. (2011). In addition, here we consider *β*‐catenin/Lef‐1 (T) acting in conjunction with either Sox10 (S) or Mitfa (M) via an AND gate to activate the production of Mitfa itself. This means that Mitfa production is activated only when either of the two AND gates involving T (one with Sox10 and the other with Mitfa) is active. The AND gate with Sox10 is activated only when the concentration of T is larger than a given threshold $\overset{¯}{T}$. A second threshold $\overset{¯}{M}$ on the concentration of Mitfa is operative also on the self‐activating loop of Mitfa.

The core of the network corresponds to what has already been modeled in Greenhill et al. (2011) with the addition of Wnt signaling. Wnt is assumed to have two components WntA (*W*_A_ in the equations above) and WntB (*W*_B_) both activating transcription factor *β*‐catenin/Lef‐1 (indicated as T in the equations). The binding and unbinding dynamics of Wnt is assumed to be fast as compared to the other network reactions, and therefore, Wnt is assumed to be equilibrated at any time. The first component WntA is present only for a transient time between 15 and 30 hpf, while the second component WntB starts at 30 hpf and remains active throughout the whole differentiation process (see Figure S4B). When these two components act together they guarantee specification and commitment of the melanocytes lineage. If only WntA is present, then we have specification but no commitment (see Figure S4C), while if only WntB is present, no specification is observed (Figure S4D). Notice that this mechanism relies on the presence of a threshold on the activation of the AND gate between Sox10 and T which prevents the production of Mitfa and therefore the activation of Mitfa's feedback loop. In order to simulate the BIO treatment, we added a correction *G*_BIO_ to the *W*_A_ and *W*_B_ components, which amounts to a time‐linear increase in Wnt when the BIO treatment is applied between *t* ^*S*^_*BIO*_ and *t* ^*E*^_*BIO*_ (times of start and end of the treatment, respectively) and is one when there is no BIO treatment. Parameter values for the model are as follows:

$$\left\{\begin{array}{c} g_{Z}=0.1\;\mathrm{nM}/h\\ g_{S}=0.3\;\mathrm{nM}/h\\ g_{M}=0.35\;\mathrm{nM}/h\\ g_{T}=1.3\;\mathrm{nM}/h\\ g_{H}=0.3\;\mathrm{nM}/h\\ g_{Ty}=3.1\;\mathrm{nM}/h\\ g_{D}=0.3\;\mathrm{nM}/h \end{array}\right.,\;\left\{\begin{array}{c} d_{Z}=0.2/h\\ d_{S}=0.3/h\\ d_{M}=0.05/h\\ d_{T}=0.6/h\\ d_{H}=0.03/h\\ d_{Ty}=0.6/h\\ d_{D}=0.1/h \end{array}\right.,\;\left\{\begin{array}{c} \Omega_{1}=1.92\;\mathrm{nM}\\ \Omega_{2}=1.43\;\mathrm{nM}\\ \Omega_{12}=1.32\;\mathrm{nM}\\ \Phi_{1}=2.0\;\mathrm{nM}\\ \Phi_{2}=0.22\;\mathrm{nM}\\ \Phi_{12}=0.1\;\mathrm{nM}\\ K_{B}=0.175\;\mathrm{nM}\\ K_{WA}=0.5\;\mathrm{nM},\\ K_{WB}=0.5\;\mathrm{nM} \end{array}\right.,\;\left\{\begin{array}{c} K_{T}=7.8\;\mathrm{nM}\\ K_{M}=0.045\;\mathrm{nM}\\ K_{M1}=1.0\;\mathrm{nM}\\ K_{M2}=1.8\;\mathrm{nM}\\ K_{M3}=1.0\;\mathrm{nM}\\ K_{Z}=6.5\;\mathrm{nM}\\ K_{S}=1.2\;\mathrm{nM} \end{array}\right.,\;\left\{\begin{array}{c} W_{A}=0.33\;\mathrm{nM}\\ W_{B}=0.25\;\mathrm{nM}\\ \overset{¯}{M}=0.01\;\mathrm{nM}\\ \overset{¯}{T}=0.75\;\mathrm{nM}\\ \Gamma_{1}=5.0/{\left(\mathrm{nM}\;h\right)}^{2}\\ \Gamma_{2}=4.5/{\left(\mathrm{nM}\;h\right)}^{2}\\ \Gamma_{12}=1.8/{\left(\mathrm{nM}\;h\right)}^{2} \end{array}\right.$$

These values are chosen in accordance with measured values from the literature (Eden et al., 2011; Jin and Liao, 1999; Schwanhäusser et al., 2011; for Sox10, Jao et al., 2013 for Mitfa) and within similar ranges as in Greenhill et al. (2011) for those unknown. We have assumed the same affinity for the two Wnt components and larger concentration for *W*_A_, but the opposite assumption (different affinity and same concentration) would lead to the same results. A slower decay rate for Mitfa than the one used in Greenhill et al., 2011 produced a better quantitative agreement with BIO treatment results.

## Supporting information


**Figure S1.** Ripley's K function analysis shows significant decrease in melanocyte organization in BIO‐treated embryos compared to DMSO‐treated embryos at 72 hpf.

**Figure S2.** Strong expression of a dntcf3 transgene results in poor melanocyte differentiation.

**Figure S3.** Ripley's K function analysis shows significant decrease in melanocyte organization when Wnt signaling is impaired in dntcf3 transgenic embryos at 72 hpf.

**Figure S4.** (A) Core GRN for melanocyte differentiation activated by Wnt signaling. (B) Schematic of WntA and WntB signaling. (C, D) Selective failure of specification and commitment. (E, F) Simulation of the core GRN according to the mathematical model (see Methods).
