# Supplementary figures and images for: An ongoing role for Wnt signaling in differentiating melanocytes in vivo
Source: Pigment Cell Melanoma Res. 2017 Mar 9;30(2):219–32. doi: 10.1111/pcmr.12568 (PMC5360516; doi:10.1111/pcmr.12568)

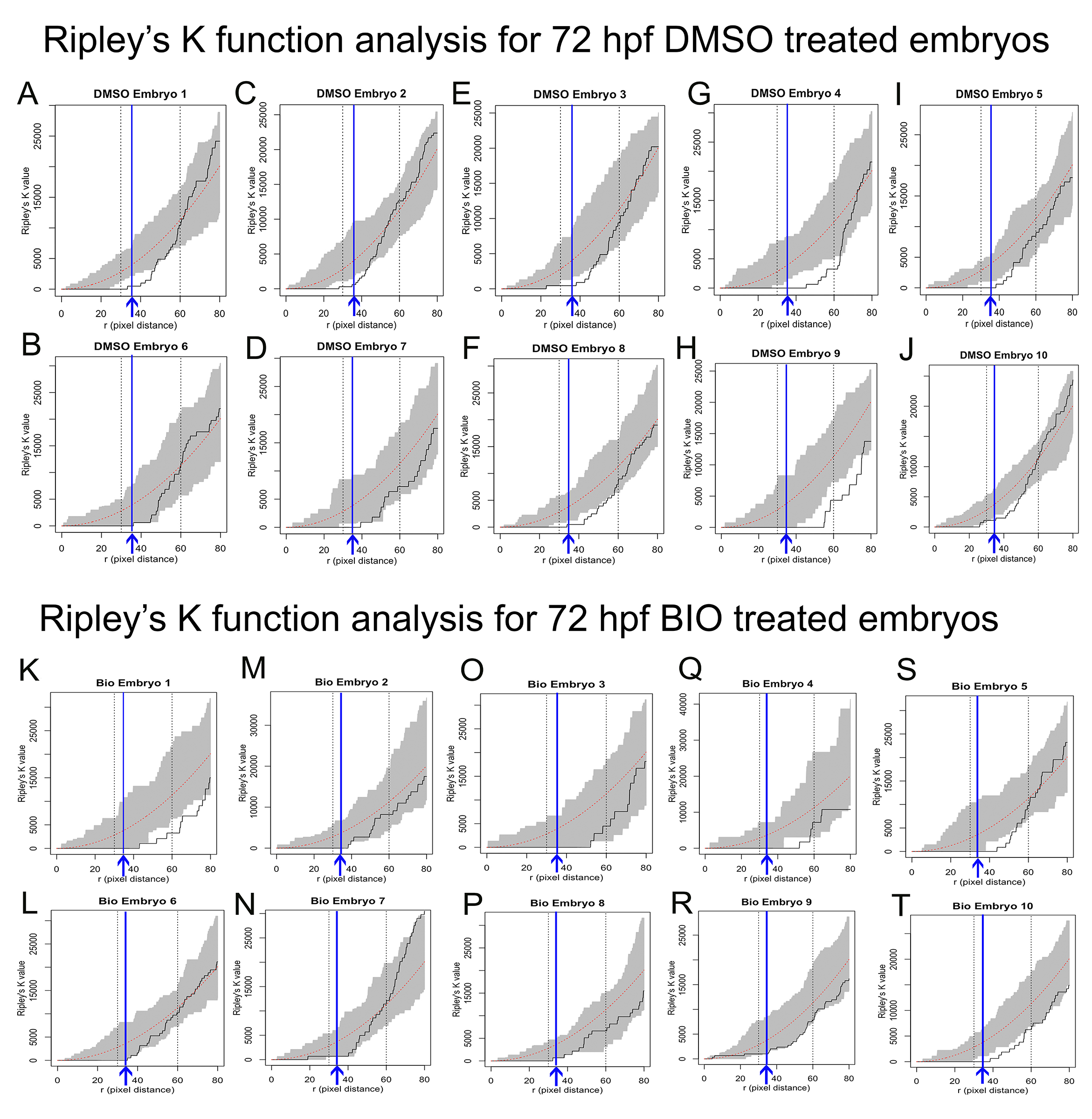

Supplement: Supplementary file 1 — Figure S1. Ripley's K function analysis shows significant decrease in melanocyte organization in BIO‐treated embryos compared to DMSO‐treated embryos at 72 hpf. [file PCMR-30-219-s001.tiff]

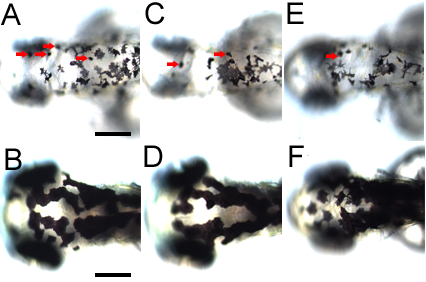

Supplement: Supplementary file 2 — Figure S2. Strong expression of a dntcf3 transgene results in poor melanocyte differentiation. [file PCMR-30-219-s002.tiff]

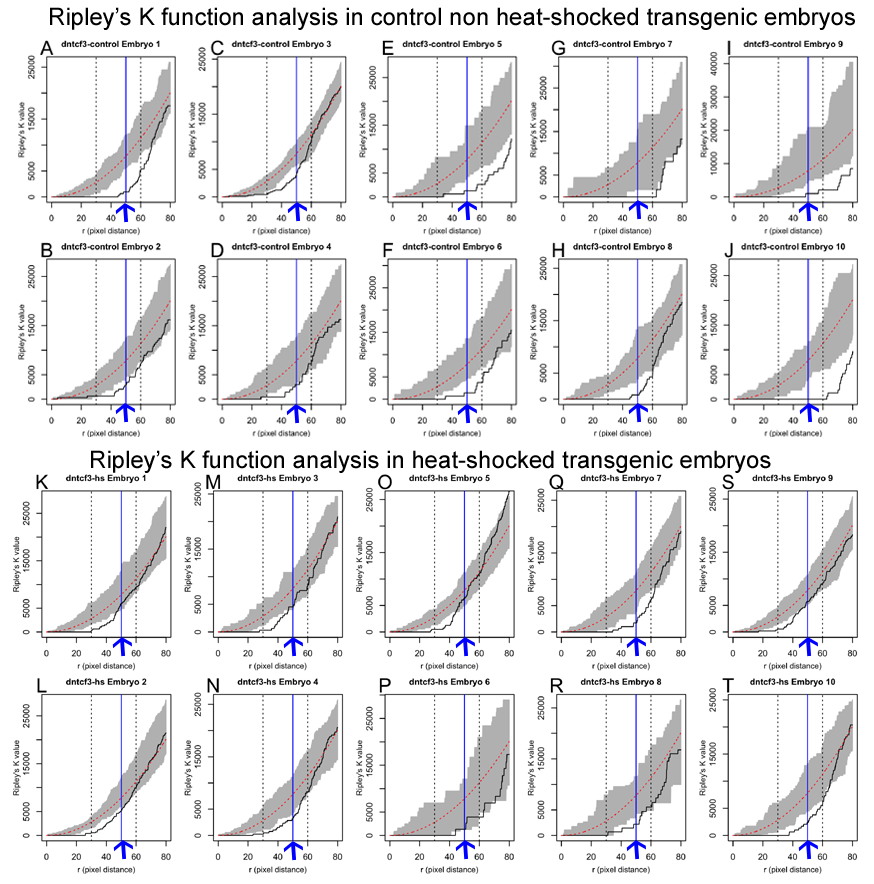

Supplement: Supplementary file 3 — Figure S3. Ripley's K function analysis shows significant decrease in melanocyte organization when Wnt signaling is impaired in dntcf3 transgenic embryos at 72 hpf. [file PCMR-30-219-s003.tiff]

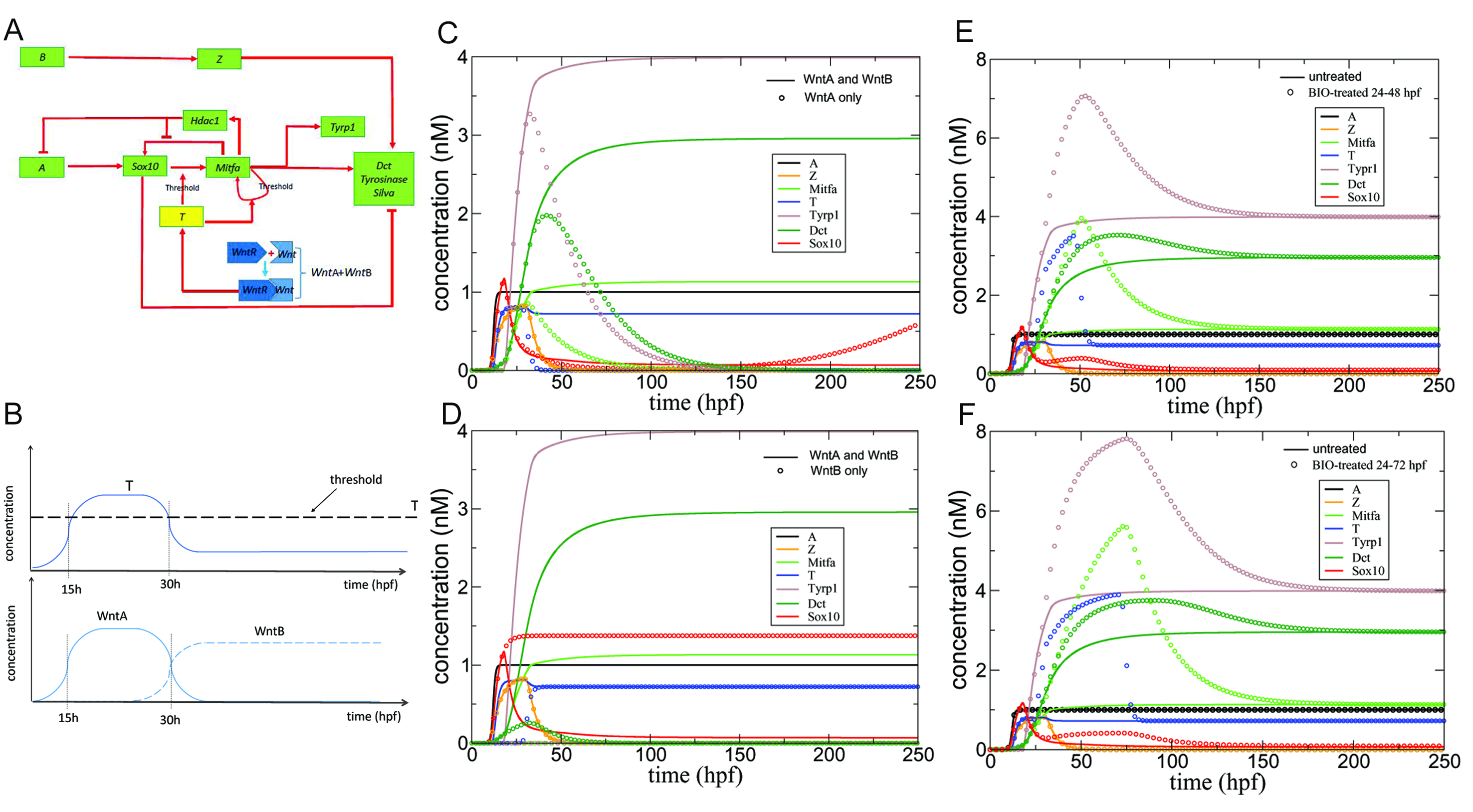

Supplement: Supplementary file 4 — Figure S4. (A) Core GRN for melanocyte differentiation activated by Wnt signaling. (B) Schematic of WntA and WntB signaling. (C, D) Selective failure of specification and commitment. (E, F) Simulation of the core GRN according to the mathematical model (see Methods). [file PCMR-30-219-s004.tiff]
